# Inequitable COVID-19 vaccine distribution and its effects

**DOI:** 10.2471/BLT.21.285616

**Published:** 2021-06-01

**Authors:** Adnan A Hyder, Maryam A Hyder, Khurram Nasir, Paul Ndebele

**Affiliations:** a Milken Institute School of Public Health, George Washington University, 950 New Hampshire Ave. NW, Washington, DC 20052, United States of America (USA).; b Barnard College of Columbia University, New York, USA.; c Center for Outcomes Research, Houston Methodist Academic Institute, Houston, USA.

The scientific community has succeeded in producing coronavirus disease 2019 (COVID-19) vaccines in record time; however, some countries are just receiving their first doses while others, such as the United States of America, have had vaccines since December 2020. The gap between available vaccines and vaccinated people raises concerns around production of vaccines, rates of vaccination, coordination between agencies, vaccine hesitancy and global health equity. We propose that the approach to COVID-19 vaccination needs to address key ethical and social justice concerns. 

We believe that four issues are key to understanding current challenges to COVID-19 vaccination. First, vaccines have historically led to positive outcomes for the poor, but not without good stewardship. Vaccines have health and economic effects that help the poor, such as improving productivity, reducing severity of disease and costs, and promoting physical and mental health.^1^^,^^2^ However, the global uptake of vaccines has often been higher in richer segments of society compared to the poor, especially in the initial stages of rollout. National governments and international actors need good stewardship to monitor and course correct such differential uptake to ensure equity in delivery.^3^ COVID-19 vaccination programmes need to be pro-poor from the beginning to reduce economic vulnerability of those at higher risk.^1^^,^^4^

Second, COVID-19 has affected some groups more than others, and COVID-19 vaccines should not do the same. The incidence of COVID-19 has been higher, and its severity more acute, in economically disadvantaged, minority and vulnerable populations.^5^ Widespread racial and ethnic disparities in COVID-19 infection and mortality is seen with disproportionately higher impact in vulnerable populations.^6^ These insights provide learning opportunities to inform an equitable vaccination programme; current disparities in procurement of COVID-19 vaccine mean that 90% of people in low- and middle-income countries are unlikely to receive the vaccine in 2021.^7^ The risk is that vulnerable groups will not receive the vaccine first, or at the right time, and will be the last to be protected, further compounding existing health inequities.

Third, COVID-19 has led to poverty, but vaccines must not do the same. The impact of COVID-19 has been seen across all spectrums of socioeconomic status, yet it affects those in the lower socioeconomic categories in many more ways. The pandemic has led to the deaths of main income earners and to loss of critical jobs, caused families to use up their savings and made the lives of the urban poor harder.^6^ The true impact of financial burden, for instance in impoverishing health expenditures is yet unknown. For example, with nearly 71% of the United States population not having the option of working from home, the financial implications of restrictions and closed workplaces will be massive in the long term.^8^

Fourth, vaccination programmes rely on real-time data and COVID-19 vaccine programmes must do the same. National vaccination programmes have always been about managing rollout at scale, and require timely reporting of supply chains, distribution and coverage indicators.^9^ For example, data on quantities of vaccines supplied by manufacturers and doses administered in each country provide critical benchmarking information for those who implement vaccination programmes and for policy-makers. Dynamic assessments of vaccine programmes are now technologically possible and must be accessible to the population; transparency helps with accountability and builds trust in the vaccine, especially in vulnerable segments of the populace.

We use the issues described above to propose the use of the term vaccine poverty to approach vaccination programmes. First, this term highlights the poverty of vaccination – those needing vaccines do not get them, or do not get them at the right time, representing a health system failure of delivery. Second, the term refers to the poverty caused by lack of vaccines; as long as some population groups remain unprotected, they are liable to contract the disease and succumb to its consequences. Third, the term refers to poverty of allocation, when needed resources have not been optimally allocated to the production and distribution of the COVID-19 vaccine.

National and global health systems and their leaders can prevent vaccine poverty. COVID-19 is still a national and international crisis and requires a strong and transparent vaccination programme. The current crisis in India heightens our fears of how vaccine poverty can affect national and local health inequity. Our call to action is to prevent and address vaccine poverty to avoid exacerbating health inequities.
